# Tumor Microenvironment Shapes Colorectal Cancer Progression, Metastasis, and Treatment Responses

**DOI:** 10.3389/fmed.2022.869010

**Published:** 2022-03-23

**Authors:** Jun Li, Dawei Chen, Minhong Shen

**Affiliations:** ^1^Department of Pharmacology, Wayne State University School of Medicine, Detroit, MI, United States; ^2^Wayne State University School of Medicine, Detroit, MI, United States; ^3^Department of Oncology, Wayne State University School of Medicine and Tumor Biology and Microenvironment Research Program, Barbara Ann Karmanos Cancer Institute, Detroit, MI, United States

**Keywords:** tumor microenvironment, colorectal cancer, metastasis, microbiota, cancer treatment

## Abstract

Colorectal cancer (CRC) is one of the most devastating diseases that accounts for numerous deaths worldwide. Tumor cell-autonomous pathways, such as the oncogenic signaling activation, significantly contribute to CRC progression and metastasis. Recent accumulating evidence suggests that the CRC microenvironment also profoundly promotes or represses this process. As the roles of the tumor microenvironment (TME) in CRC progression and metastasis is gradually uncovered, the importance of these non-cell-autonomous signaling pathways is appreciated. However, we are still at the beginning of this TME function exploring process. In this review, we summarize the current understanding of the TME in CRC progression and metastasis by focusing on the gut microbiota and host cellular and non-cellular components. We also briefly discuss TME-remodeling therapies in CRC.

## Introduction

Colorectal cancer (CRC) is one of the most common malignancies for both males and females, ranking in the top three for both estimated incident cases and deaths (1). WNT signaling hyperactivation induced by mutational inactivation of the Adenomatous Polyposis Coli (APC) accounts for most CRC cases in patients (2). In addition, other somatic mutations, such as the tumor suppressor P53 and oncogenic pathway KRAS are commonly observed in CRC (3, 4). Although these tumor cell-autonomous pathways significantly contribute to CRC progression and metastasis, therapeutic interventions that target these pathways have achieved limited success in patients. Chemo- and radio-therapies, which have substantial adverse effects, are still commonly used for CRC patients, especially for those at advanced stages. Therefore, therapeutic strategies with better efficacy and less toxicity are urgently needed.

Accumulating evidence indicates that non-cell-autonomous pathways, especially signaling pathways of the tumor microenvironment (TME), are significantly involved in CRC progression and metastasis, either by promoting or inhibiting the process. TME refers to a special biological environment formed by malignant cells, non-malignant cells and their secreted components as summarized in a previous review (5). Given the pivotal roles of the TME in CRC progression and metastasis, exploration of the mechanisms underlying the interplay between TME remodeling and CRC development have attracted substantial attention over the last decade. Great progress has been made in this field, which has dramatically advanced our knowledge about the TME and CRC. New insights about CRC treatment have also been elucidated. However, a complete understanding of the TME in CRC progression and metastasis has yet to be unfolded.

The TME dynamically changes with enormous complexity throughout cancer progression. In general, it is composed of cellular and non-cellular components, which have distinct functions but also collaborate with each other during cancer progression and metastasis (6). In this review, we summarize our current understanding of the roles of the TME in CRC progression and metastasis by focusing on the components in both primary sites and distant metastatic organs. We also briefly discuss the therapeutic insights on remodeling the TME in CRC.

## TME in Primary Sites

The main components of the TME are the extracellular matrix (ECM) and its cellular partners, including immune cells, endothelial cells and fibroblasts. In addition to these common components, intestinal cells are also in close contact with a large population of microorganisms referred to as the gut microbiota (7). In this section, we summarize the roles of these common components as well as a few key microorganisms in CRC progression and metastasis.

## Microbiota: Friend and Foe

**FIGURE 1:**
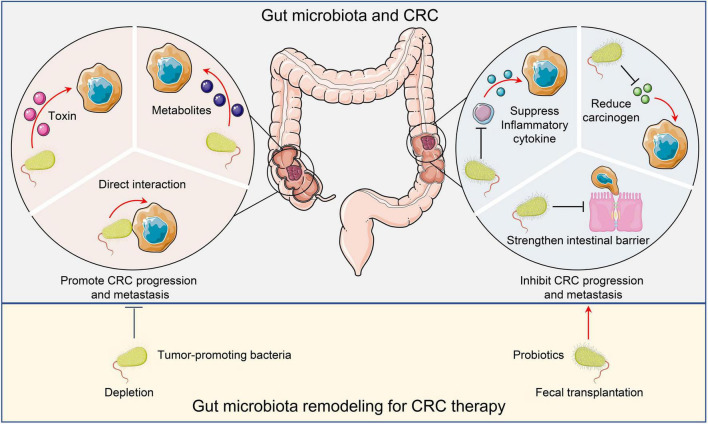
Microbiota and CRC. Tumor promoting microorganisms facilitate CRC progression and metastasis by directly interacting with malignant cells, secreting toxins, or producing metabolites to activate oncogenic pathways. Conversely, probiotics inhibit CRC cancer progression and metastasis by suppressing inflammation, reducing carcinogens, and maintaining intestinal barrier integrity. Remodeling the gut microbiota by depleting tumor-promoting bacteria or administering probiotics may have therapeutic potential. CRC, Colorectal cancer.

The critical roles of microorganisms in our gastrointestinal tract have long been known. On one hand, the microorganisms help with digestion and maintain homeostasis. On the other hand, they establish and promote disease progression (Figure 1). One of the most famous examples is *Helicobacter pylori* (*H. pylori*). The positive correlation between *H. pylori* infection and gastric cancer has been well recognized (8). Compared to the stomach, the colon has an even more diverse microbiota, which is also significantly involved in maintenance of homeostasis and progression of diseases.

### Microorganisms That Promote CRC Progression and Metastasis

Trillions of microorganisms reside in the gut. Some of these microorganisms are potentially pathogenic. Several species, such as *Enterococcus faecalis (E. faecalis)*, *Streptococcus gallolyticus* subsp. *gallolyticus (Sgg)*, *H. pylori*, *Bacteroides fragilis (B. fragilis)*, *Clostridium septicum (C. septicum)*, *Escherichia coli (E. coli)*, and *Fusobacterium nucleatum (F. nucleatum)* have been reported to elevate colorectal carcinogenesis (9). *E. faecalis* produces extracellular superoxide, induce DNA damage and genomic instability in colonic epithelial cells, and activate macrophages to produce 4-hydroxy-2-non-enal, thereby promoting colon cancer in mice (10). Another study revealed that *E. faecalis* infection also prevents intestinal epithelial cells from activating protective TGF-β/Smad signaling, and thus, promote CRC progression (11). The association between *Sgg* and CRC has been well recognized as well. *Sgg* infection activates a few oncogenic pathways such as Wnt/β-catenin, c-Myc, and PCNA, and therefore, promotes CRC (12). Interestingly, the unique CRC TME also elevates *Sgg* colonization, which disturbs the ecological balance in the colon and further exacerbates CRC (13). In addition to gastric cancer, the association between *H. pylori* infection and CRC has also been reported (14), but the mechanism remains elusive. One possibility is that the vacA toxin produced by *H. pylori* results in cell proliferation dysregulation, and thus, induces CRC initiation and progression (15). Similarly, *B. fragilis* toxin (BFT) activates Wnt and NF-κB signaling, leading to DNA damage and the initiation and promotion of CRC (16). The prevalence of the BFT gene in CRC patients has been confirmed (17). While *C. septicum* does not appear to initiate CRC, the α-toxin produced by *C. septicum* enhances dissemination and circulation of tumor cells (18). Moreover, the tumorigenic role of *E. coli* in CRC has been extensively studied. *E. coli* produces colibactin, a genotoxin, to elevate CRC progression and metastasis through distinct mechanisms: enhancing tumor cell proliferation (19), and promoting pro-tumoral activity of immune cells (20). Unlike other bacteria that utilize toxins to promote CRC progression and metastasis, *F. nucleatum* directly activates Wnt/β-catenin modulator Annexin A1 through interaction with malignant cells, and thus, promotes CRC progression (21).

In addition to the microorganisms themselves, microbial metabolites, such as short-chain fatty acids (SCFAs), secondary bile acids, and glucuronidase, produced after the destruction of intestinal microecology also affect the development of colorectal cancer. SCFAs are major bacterial metabolites that play multiple roles in homeostasis as well as pathogenesis. Previous study indicates that Butyrate (a type of SCFA), promotes CRC tumorigenesis by provoking cellular senescence (22). Similarly, studies have shown that OSTβ, which is an important subunit of a bile acid export transporter OSTα-OSTβ, is significantly downregulated in CRC, suggesting an important role of bile acid in CRC development (23). Another study indicated that deoxycholic acid (another secondary bile acid) promotes the development of colorectal tumors in rats that exposed to azomethane (carcinogen) (24). In addition, the activity of glucuronidase in feces of patients with colorectal cancer is higher than that of normal people. Inhibition of glucuronidase activity was found to effectively reduce the number of tumors in a mouse model with colorectal cancer (25). Moreover, fucoxanthin can prevent colon cancer by inhibiting the activity of glucuronic acid (26). Collectively, these studies fully illustrated the critical roles of microbial metabolites in CRC progression and metastasis.

### Probiotics

Conversely, gut microorganisms can repress CRC progression and metastasis. Different probiotics inhibit CRC *via* distinct mechanisms. For instance, probiotics could reduce DNA damage or downregulate inflammation to inhibit CRC (27). Yue et al. reported that administration of the probiotic strain *Lactobacillus plantarum* YYC-3 prevents CRC by reducing inflammation. Mechanistically, *L. plantarum* YYC-3 suppresses NF-κB and Wnt signaling in tumor cells to inhibit inflammatory cytokine production (28). In another study, tumor size decreased upon administering probiotic strain *C. butyricum* and 1,2-two hydrazine hydrochloride in a CRC mouse model (24). The treatment reduced Th2 and Th17 cells in tumors, and therefore, decreased tumor infiltrated CD4^+^ and CD8^+^ T lymphocytes. Consequently, this inhibited the secretion of inflammatory factors such as NF-κB and IL-22, which impeded cell cycle progression and enhanced tumor cell apoptosis (29). In human subjects, probiotic strains *Lactobacillus acidophilus* 145 and *Bifidobacterium longum* 913 prevented DNA damage in human colon tumor cells (30). Specifically, healthy volunteers were given standard yogurt or probiotic yogurt that contained *L. acidophilus* 145 and *B. longum* 913. Fecal water was collected to test the genotoxicity on human colon cancer cells HT29clone19A. This revealed that the probiotics reduced the risk of colon cancer by inhibiting carcinogen-induced DNA damage (30).

Instead of directly targeting tumor cells, the reduced inflammation induced by probiotics could also strengthen the intestinal barrier and suppress metastasis. Tight junction proteins play important roles in intestinal integrity and permeability. The probiotic strain *Lactobacillus* enhances the integrity of tight junctions and reduces intestinal permeability. Treatment with *Lactobacillus rhamnosus* GG and *Lactobacillus reesei* ZJ617 helped to reduce oxidative stress and inflammation, which led to increased expression of tight junction proteins, thereby restoring intestinal barrier function (31). In accordance with this notion, *Lactobacillus* inhibited colon cancer in the mouse model (32).

Moreover, probiotics could also reduce the production of intestinal carcinogens or carcinogenic metabolites to prevent the occurrence of CRC. Studies have shown that *L. rhamnosus* achieves anti-tumor and anti-genotoxic effects by binding 1-methyl-3-nitro-1-nitrosoguanidine. It also inhibits the production of toxic carcinogens such as glucuronidase and glucosidase in the intestinal tract (33, 34).

## Cellular Components in the TME

**FIGURE 2:**
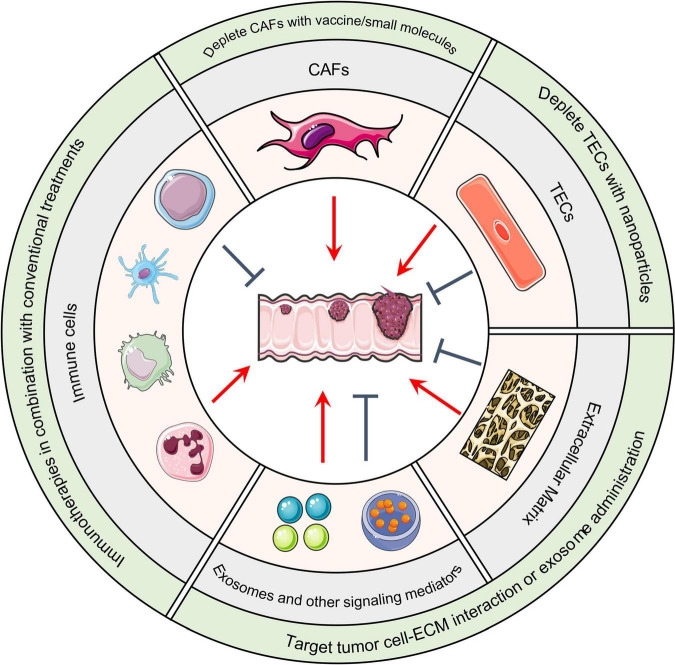
Host TME components and CRC. Cellular and non-cellular TME components can either promote or suppress tumor formation based on the situation. Immunotherapies that remodel the immune cell population in combination with conventional treatments such as chemo- and radiotherapies are under evaluation. DNA vaccines, small molecules, or nanoparticles that deplete CAFs or TECs are therapeutic strategies under development. In regard to altering non-cellular components of the TME, integrin antagonist treatments that block the interaction between malignant cells and the ECM has achieved considerable success in pre-clinical models. Administration of anti-tumor exosomes has achieved similar success. TME, Tumor microenvironment; CRC, Colorectal cancer; CAFs, Cancer-associated fibroblasts; TECs, Tumor-associated endothelial cells; ECM, Extracellular matrix.

In addition to microorganisms, the primary CRC tumors have cellular components within its microenvironment. In this section, we discuss the tumor promoting or suppressing roles of immune cells, cancer-associated fibroblasts, and endothelial cells (Figure 2).

### Immune Cells: Lymphocytes

Tumor infiltrating lymphocytes (TILs) are a group of immune cells in the TME. They are composed of a variety of T cell subpopulations such as CD4^+^T cells, CD8^+^T cells, B cells, and NK cells. TILs can be involved in tumor immune evasion as well as tumor recognition, destruction, and elimination (35). The function of these traditional TILs in CRC has been thoroughly discussed (36, 37), therefore we did not redundantly summarize their function in this review. However, in addition to these traditional lymphoid cells, another subset known as innate lymphoid cells (ILCs) have been recognized and their function has been appreciated. For example, ILCs located on the mucosal surface of the intestine enhance the immune response, maintain mucosal integrity, and promote lymphatic organ formation (38). In addition to this normal context, single-cell transcriptomic analysis has revealed that signaling lymphocytic activation molecule family member 1 (SLAMF1) is selectively expressed on CRC tumor-specific ILCs. SLAMF1-high ILCs could serve as an anti-tumor biomarker in CRC (39). Another study reported that a subset of ILCs, ILC3, helps regulate the balance between the immune system and gut microbes to prevent CRC (40).

### Immune Cells: Macrophages

Macrophages, which are a subset of myeloid cells, were found to inhibit the proliferation, migration, invasion, and metastasis of CRC. Meanwhile, they are also indispensable in cancer progression and treatment resistance (41). In general, macrophages can be classified as classical (M1) or alternative activated (M2) subtypes. During normal immune responses, most macrophages differentiate to the M1 phenotype, which inhibits CRC and are involved in Th1 cytokine responses upon pathogen challenging (42). However, M2 macrophages were found to promote tumor progression through multiple pathways: (1) produce epidermal growth factor and fibroblast growth factor-1 to foster tumor cells, (2) secrete vascular endothelial growth factor A to promote angiogenesis, and (3) release matrix metalloproteinases to promote invasion. In addition, M2 macrophages inhibit immune responses by producing immunomodulators such as IL-10, IL-6, and TGF-β1. They also induce immunosuppression by recruiting Th2 and Treg through secretion of anti-inflammatory chemokines such as CXCL17, 22, and 24. All these factors promote tumor progression (43–46).

### Immune Cells: Dendritic Cells

Dendritic cells (DCs), which also differentiate from myeloid progenitors, are key cells in the adaptive immune response and are essential for T cell-mediated cancer immunity. Infiltration of the TME by normal mature DCs is correlated with a favorable prognosis in ovarian cancer (47, 48). In the TME, abnormal DC formation can be generally grouped into three causes: abnormal differentiation of precursors leading to a decrease in the number of cells, phenotypic changes inducing immune tolerance, and inhibition of cell maturation leading to functional abnormalities. Abnormal DCs have insufficient antigen recognition and cannot provide adequate costimulatory signals for T cell activation. According to clinical studies, infiltration of the TME by normal mature DCs is correlated with a favorable prognosis in ovarian cancer (49). Meanwhile, tumor cells and DCs have profound interplay in TME. Loss or down-regulation of the epithelial-specific transcription factor Ese-3 gene expression in colon cancer cells results in impaired DC maturation. Moreover, abnormal DCs could promote tumor cell proliferation (50, 51). The functional defects of DCs accounts for the low efficiency of the anti-tumor specific immune response. As such, intervention to reverse DC functional defects is a potential strategy for the treatment of CRC (52).

### Immune Cells: Neutrophils

Neutrophils, another subset of the myeloid population, could both promote and suppress tumor formation and progression. On one hand, they have defensive functions against tumor; on the other hand, neutrophils in TME may support tumor progression. Recent study indicated that neutrophils restrict the microbiota in tumors to reduce CRC progression and metastasis in mouse models (53). Depletion of neutrophils disrupts the gut microbiota and leads to an increased number of bacteria that secrete IL17, which promotes tumor growth and progression (53). Meanwhile, accumulating evidence suggests that neutrophils stimulate CRC progression and metastasis through the CXCL1/CXCR2 chemokine axis (54) and remodel the ECM microenvironment by producing matrix metalloproteinase MMP9 (55).

### Cancer-Associated Fibroblasts

Cancer-associated fibroblasts (CAFs), the most numerous cellular components of the TME, promote tumor angiogenesis, cell proliferation, migration, and metastasis *via* multiple mechanisms. CAFs secrete various chemokines and cytokines that interact with tumor cells to promote CRC progression (56, 57). In addition, exosomal miRNAs from CAFs act as intercellular signaling molecules to modulate pathways such as KRAS, MYC, and TGF-β, all of which are involved in tumor progression (58, 59). For example, exosomal miRNA-17-5p from CAFs directly targets RUNX family transcription factor 3 (RUNX3). RUNX3 interacts with the proto-oncogene MYC and binds to the promoter of TGF-β1, thereby activating the TGF-β signaling pathway. The RUNX3/MYC/TGF-β1 pathway promotes CRC proliferation, chemoresistance, and metastasis (60). Interestingly, this pathway also activates CAFs *via* positive feedback and further accelerates CRC progression and metastasis (60). Aside from the aforementioned mechanisms, CAFs also directly interact with tumor cells to accelerate cancer progression and metastasis (61–63).

Besides targeting tumor cells, CAFs may remodel other stromal cells to promote CRC. Emerging evidence revealed that CAFs reshape immune cell populations in the TME. Zadka et al. found that CAFs are negatively correlated with tumor-infiltrating lymphocytes (64). Consistently, CAFs were found to recruit monocytes and promote M2 polarization of macrophages by upregulating adhesion molecules such as ICAM-1 or VCAM-1, or through the IL-8/CXCR2 pathway. The tumor associated macrophages then synergize with CAFs to suppress immunosurveillance (65, 66). In addition, CAFs promote endothelial cells to release vascular endothelial growth factor (VEGF), leading to consequential angiogenesis (67).

As one of the major players of the ECM organization, CAFs deposit numerous ECM proteins involved in cancer progression (68, 69). Previous study indicated that CAFs produce HGF and ECM glycoprotein tenascin-C to promote CRC invasion (70). Moreover, CAFs were found to secret Activin A to increase the stiffness of the ECM, and thus, promote CRC progression and metastasis (71).

### Endothelial Cells

Endothelial cells are the main components of vascular vessels. Angiogenesis is crucial for cancer progression by supplying oxygen and nutrients while removing toxic metabolites. Angiogenesis also provides a conduit for tumor cell dissemination and metastasis (72, 73). Tumor-associated endothelial cells (TECs) in the TME were found to produce vascular endothelial growth factor receptor (VEGFR) and other growth factor receptors such as EGFR to enhance angiogenesis (74). Additionally, TECs promote CRC progression and metastasis through other pathways. Markedly, TECs produce a soluble form of Jagged-1, which activates Notch signaling and promotes the cancer stem cell phenotype in CRC cells (75). Furthermore, TECs could express adhesion molecules, such as E-selectin to facilitate CRC invasion and metastasis (76).

Similar to CAFs, TECs modulate immune cell populations. For instance, TECs express FasL to eliminate CD8^+^ T cells and enhance immune evasion of cancer cells (77). The E-selectin expressed by TECs could also attract more neutrophils to establish an immunosuppressive TME. A comprehensive endothelial cell-derived transcriptome analysis was performed recently. Consistent with previous findings, a set of hub genes such as *SPARC*, *COL1A1*, *COL1A2* and *IGFBP3* is positively correlated with immune-inhibitory markers of various immunosuppressive cells, including TAM, M2 macrophage, and Tregs. T cell exhaustion was also identified (78).

Unlike CAFs that mainly behave as a tumor promoter within the TME, a few subsets of TECs could inhibit CRC progression. For example, Apelin induces chemokine CCL8 expression in TECs, and the increased CCL8 expression may enhance CD8^+^ T cell infiltration in TME, and thus, suppress CRC progression (79). The quiescent-inducing activity of endothelial cell-derived SPARCL1 has also been shown to potentially contribute to Th1-TME-related vascular quiescent micromigration in colorectal cancer. SPARCL1 promotes the antitumor microenvironment by inducing cell immobilization and limiting blood vessel formation (80).

## Non-Cellular Components

In addition to the cellular components discussed above, the ECM built by the cellular components serve as a scaffold and is an essential component of the TME. Small molecules and vesiculas secreted by these cells facilitate signaling transduction and play essential roles in CRC progression and metastasis.

### Extracellular Matrix

The ECM contains proteins secreted by both malignant and non-malignant cells in the TME. These include collagen, fibronectin, integrin, elastin, microfibrillin, and proteoglycans, all of which support neighboring cells structurally and biochemically (81). Collagen, a major ECM protein, is a diverse protein family with at least 28 members (82). The tumor promoting effects of collagen content and distribution have been extensively studied (83). For instance, collagen has been found to promote the CRC stemness and metastasis by targeting the integrin/PI3K/AKT/Snail pathway (84). Collagen type V α2 (COL5A2) has been found to correlate with poor prognosis in CRC (85). Similarly, fibronectin also promotes CRC progression and is correlated with poor prognosis in patients (86, 87).

There is no doubt that each individual ECM protein contributes to CRC progression and metastasis in distinct ways, which we are unable to cover in detail. However, as a whole, the overall amount of ECM protein deposition contributes to the stiffness of the TME (88). Correspondingly, increased ECM stiffness is a hallmark of CRC progression and metastasis (71, 89, 90).

Conversely, the ECM can impede cancer progression as well. For example, collagen type IV may suppress CRC invasion (91). Furthermore, because the ECM is located in the stroma between the basement membrane and interstitial space, it acts as a natural barrier for tumor cell proliferation, differentiation, and metastasis (92).

### Other Secreted Components

The direct physical cellular interactions in the TME are undoubtedly critical for signaling transduction. However, molecules, such as TGF-β, Wnt, other metabolites, and secreted exosomes also mediate cell communication, and therefore, are involved in cancer progression and metastasis. Although TGF-β may suppress CRC in certain contexts (93), the tumor promoting roles of TGF-β and Wnt have been well recognized and extensively validated (94, 95).

In addition to these signal mediating small molecules, numerous exosomes secreted by both malignant and non-malignant cells facilitate signal transduction. MicroRNAs (miRNAs) are one of the main contents of these secreted exosomes. Exosomal microRNAs play pivotal pro- and anti-tumoral roles in CRC progression and metastasis. They could also serve as biomarkers of CRC progression in patients (96, 97). On one hand, exosomal miR-21 promotes CRC cell proliferation, invasion, and therapy resistance (98). On the other hand, exosomal miR-379 secreted by cancer cells suppresses CRC cell proliferation and migration (99).

## TME in Distant Metastatic Organs

**FIGURE 3:**
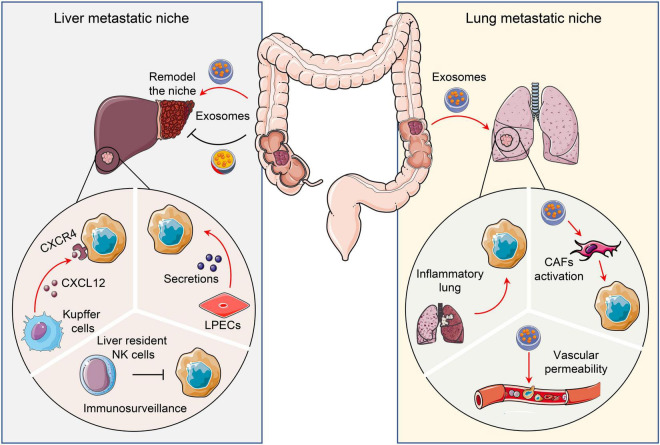
Pro- and anti-tumor niches in liver and lung. Tissue specific cells attract or restrain CRC metastatic colonization and outgrowth *via* distinct mechanisms. To illustrate, liver and lungs form a pro- or anti-tumor niche *via* tumor secretomes, including chemokines and exosomes. CRC, Colorectal cancer; CAFs, Cancer-associated fibroblasts; LPECs, Liver parenchymal endothelial cells.

During late-stage development of CRC, tumor cells metastasize to distant organs. The tumor cells together with stromal cells create a microenvironment to either foster or restrain the outgrowth of the metastatic tumors. As such, the TME in metastatic organs harbors common cellular and non-cellular components as discussed above. However, the metastatic organs could also have their own unique stromal components that are involved in CRC colonization and outgrowth (Figure 3).

## Liver Metastases

Around 20-25% of patients initially diagnosed with colorectal carcinoma present with liver metastases (100). Of these, 80–90% have multiple organ metastasis, including liver, and 50% have exclusively liver metastasis (101). This is partially due to the liver being proximal to the colon and their intimate connection by portal circulation. A large number of tumor cells upon extravasation from primary sites could directly disseminate to liver *via* blood circulation. Meanwhile, the liver may create a unique microenvironment to attract and foster tumor colonization and outgrowth. To illustrate, Kupffer cells, which are a set of specialized cells localized in the liver, highly express CXCL12 to attract CXCR4-expressed CRC cells. This facilitates tumor cell colonization and outgrowth (102). Likewise, liver parenchymal endothelial cells (LPECs) activate the HER3-AKT pathway in tumor cells *via* paracrine signaling to promote CRC liver metastasis (103). Conversely, liver cells could also restrain metastatic colonization. For example, liver resident NK cells provide immune surveillance and eliminate tumor cells that disseminate to the organ. Harmon et al. found that reduction of these liver resident NK cells significantly promotes CRC liver metastasis (104).

Pro- or anti-metastasis communication between CRC cells and liver stromal cells could occur even before the residence of tumor cells in the liver. Tumor cells reshape the liver microenvironment through secretomes before their arrival. Shao et al. found that CRC secreted exosomes are enriched with microRNA-21-5p (miR-21). These induce liver macrophage polarization toward an interleukin-6 (IL-6)-secreting proinflammatory phenotype. As a result, the proinflammatory niche promotes liver metastasis (105). However, exosomal Angiopoietin-like protein 1 (ANGPTL1) attenuates CRC liver metastasis. Mechanistically, exosomal ANGPTL1 is taken up by Kupffer cells in the liver, which alters their secretion patten by targeting the JAK2-STAT3 pathway. The altered Kupffer cells decrease MMP9 expression and prevent liver vascular leakiness, and consequently, suppress liver metastasis (106).

## Lung Metastases

The lungs are the second most common metastatic site of CRC after the liver. Commonly, lung metastasis occurs with an incidence of 10–15% in patients after radical resection of CRC (107). Previous study found that the activation of NF-κB signaling in CRC cells enhances TNF-α production of host hematopoietic cells. This results in a pro-inflammatory microenvironment within the lungs that promotes CRC lung metastasis (108). Similar to the liver, the secretomes of tumor cells could remodel the lung microenvironment to favor CRC lung metastasis. Exosomal miR-25-3p secreted by CRC cells facilitates metastasis by promoting vascular permeability and angiogenesis by targeting endothelial cells in the lung. Mechanistically, it targets KLF2 and KLF4 to regulate the expression of VEGFR2, ZO-1, occludin and Claudin5 (109). In addition, CRC primary tumors release integrin beta-like 1 (ITGBL1)-rich extracellular vesicles (EVs) to stimulate the TNFAIP3-mediated NF-κB signaling pathway and activate resident fibroblasts in distant organs (110). These activated fibroblasts produce pro-inflammatory cytokines such as IL-6 and IL-8 to create a pre-metastatic niche in the lung (110).

## Metastases in Other Tissues

In addition to the liver and lungs, CRC cells could metastasize to the peritoneum, brain, and ovary. Five to ten percent of CRC patients are diagnosed with peritoneal metastases. This increases to 20-50% in recurrent CRC patients (111). Peritoneal metastasis can originate from preoperative tumor cell dissemination, intraoperative trauma-induced tumor cell shedding, metastatic lymph node rupture, lymphatic tumor thrombus rupture, or surgical field hemorrhage. All of which induces the implantation of free cancer cells in the peritoneum (112, 113). The peritoneal microenvironment is composed of a variety of cellular components, such as human peritoneal mesothelial cells (PMCs), peritoneal fibroblasts (PFBs), peritoneal macrophages (PMs), and adipocytes, which create a suitable soil for peritoneal metastasis. For example, the expression of intercellular adhesion molecule-1 (ICAM-1) and vascular adhesion molecule-1 (VCAM-1) in CRC cells can increase their adhesion to PMCs (114).

Two to twelve percent of CRC patients develop brain metastasis (115). Significantly, the blood-brain barrier is a natural fence that separate the brain with the rest of the body. It creates a unique brain microenvironment. It also serves as the first line of defense toward tumor cells dissemination to the brain. Nevertheless, CRC tumor cells can still metastasize to the brain. Previous studies revealed that nitric oxide (NO) is a crucial mediator of anti-tumor properties of microglia in the TME of the brain (116). However, metastatic CRC cells may suppress the cytokine-induced NO production in cerebral endothelial cells to inhibit the activation of microglia (117), and consequently, promote the outgrowth of CRC brain metastases.

Ovarian metastasis of colorectal cancer is relatively rare and usually occur in younger patients (118). Notably, the ovaries are rich in lymphatic vessels and there are lymphatic junctions between the colorectum and ovaries bilaterally. Studies suggested that CRC cells take advantage of the lymphatic vessel rich microenvironment to invade the lymphatic system and subsequently metastasize into the ovaries (119, 120).

## Therapeutic Implications by Targeting TME

Thanks to the development of cancer screening and therapeutic strategies, the number of CRC deaths has been declining over the last few decades. However, at least 50,000 patients may die from CRC in 2022 within the United States. It is still a devastating disease, and curative therapeutic interventions are urgently needed. Given the critical functions of the TME in CRC progression and metastasis and our accumulating knowledge on this subject, new insights about CRC therapy by targeting TME are emerging (Figures 1, 2).

As discussed above, the gut microbiota plays essential roles in CRC progression and metastasis. Manipulation of the gut microbiota could hold clinical implications for CRC patients. Intratumor Gammaproteobacteria residing in the TME metabolize the chemotherapeutic drug gemcitabine (2′,2′-difluorodeoxycytidine) into its inactive form, 2′,2′-difluorodeoxyuridine. Antibiotic-induced depletion of this bacteria enhanced the gemcitabine-mediated chemotherapy response in a CRC mouse model (121). Alternatively, fecal microbiota transplantation (FMT) may also be employed to prevent CRC in patients with dysbiosis (122). A FMT procedure could help eradicate procarcinogenic *E. coli* (114). Encouraged by these pre-clinical findings, a few clinical trials on manipulating gut microbiota in CRC patients are ongoing (123).

Immunotherapies such as immune checkpoint blockade therapy, Chimeric antigen receptor (CAR) T-cell therapy, and T-Cell Receptor (TCR) Therapy have achieved considerable success in several cancer types (124). Immune checkpoint blockade therapy such as PD-1 and CTLA-4 inhibitors have been approved as first or second line of treatment for metastatic CRC patients with microsatellite instability-high (MSI-H). Unfortunately, treatments in MSI-H CRC patients usually only extend survival rate rather than induce complete regression of metastatic lesions. Even worse, the majority (>85%) of CRC patients with microsatellite instability-low (MSI-L) are not eligible for this therapy. Immunotherapy in combination with other conventional treatments, such as chemotherapy, radiotherapy, and targeted therapy are currently being tested in order to overcome these obstacles and to encompass a broader CRC population with better efficacy (125).

Given the central prooncogenic roles of CAFs, therapies that target CAFs have been emerging. Fibroblast activation protein (FAP) is a commonly used marker for CAFs and serves as an ideal target for CAFs eradication. Pre-clinical studies indicate that administration of DNA vaccine that targets FAP effectively depletes CAFs in the TME, and consequently suppresses CRC progression and metastasis (126). Furthermore, CAF depletion by small-molecule dipeptidyl peptidase inhibitor PT-100 significantly enhanced the efficacy of chemotherapy in CRC mouse models (127). In addition, a small chemical compound, Atractyloside, was also found to inhibit CRC metastasis by targeting CAFs (128). Collectively, all these preclinical studies suggest that CAFs in the TME could be a potential target for CRC.

One of the most common therapies for CRC is to target VEGF and prevent endothelial cell-mediated angiogenesis. However, this method has considerable adverse side effects and limited benefit to patients because it targets both TECs and normal endothelial cells. Nanoparticles that could selectively recognize and deplete TECs have been developed and tested in pre-clinical models (129).

Aside from targeting or remodeling cellular components in the TME, manipulating non-cellular components could have therapeutic potential as well. As mentioned above, fibronectin is one of the major components of the ECM that promotes CRC progression and metastasis. ATN-161, which is a non-RGD-based pentapeptide (PHSRN) derived from the fibronectin synergy region, inhibits breast cancer progression and metastasis by antagonizing fibronectin (130). It was shown that ATN-161 suppresses metastatic CRC progression and sensitize it to chemotherapy in mouse model (131). Similarly, HM-3, which is another integrin RGD domain containing peptide, remodels the ECM and achieves impressive anti-tumor efficacy in CRC xenograft models (132). In addition to remodeling the ECM, manipulating other cell-secreted TME components have attracted attention. Ascites-derived exosomes (Aexs) together with the granulocyte-macrophage colony-stimulating factor (GM-CSF) achieved encouraging outcomes in a phase I clinical trial with advanced CRC patients (133).

## Conclusion and Perspective

The TME has been recognized as a key player in tumor progression and metastasis in CRC. All the components from both microorganisms and the host are significantly involved in this process. Although each component has distinct roles during CRC progression and metastasis, most of them behave like a double-edged sword, which could both promote or inhibit tumor progression depending on the specific context. Moreover, the TME is highly dynamic during CRC progression and metastasis. The properties and abundance of each TME component could be significantly altered during tumor progression and treatments. In this review, we only covered a few common components and discussed their functions mainly from a tumor-promoting point of view. Of note, polymorphic microbiomes are one of the hallmarks of cancer. Many organs other than the colon, including the liver and lungs may have distinct microbiomes (134). Here, we only discussed the gut microbiota and their potential function in primary tumor sites. However, as mentioned above, both microorganisms and host non-malignant components dramatically contribute to the initiation and progression of CRC.

Cancer metastasis is composed of a serial of complex process, from extravasation from primary sites to the eventual colonization at distant organs (135). Although a considerable number of CRC patients have metastatic disease at the time of diagnosis or recurrency, only a small fraction of the disseminated cancer cells are capable of successful metastases (136). Disseminated tumor cells encounter tremendous stresses from the TME during metastasis (137). Each component of TME contributes to this metastatic cascade. The specific components of the TME and their roles during the metastatic cascade have not been covered here as it has been comprehensively discussed in our previous review (137). In this review, we mainly focused on established metastases. Moreover, we emphasized unique metastatic organ components rather than restated the common TME components (such as CAFs and ECM) of primary tumors. Collectively, unique stromal cells of various tissues promote or restrain metastatic outgrowth with distinct pathways as discussed above.

With the accumulating knowledge about TME in CRC progression and metastasis, promising therapeutic strategies that modulate TME are emerging. As mentioned earlier, many researchers have confirmed that better anti-tumor effects can be achieved through remodeling of the TME. In addition to the beneficial effects in pre-clinical studies, observations in clinical trials have shown the great potential of TME remodeling to improve the therapeutic effect of drugs.

However, due to the dynamic changes of the TME during cancer progression and metastasis, a detailed, functional dissemination of every TME component at each given tumor stage is essential. This knowledge would help us stratify CRC patients that could benefit from a specific TME remodeling therapy, and more importantly, suggest the best treatment window. Furthermore, monotherapy is usually insufficient because tumors, especially metastatic tumors, have the tendency to develop resistance. In order to minimize treatment resistance, combining TME remodeling strategies with other promising therapeutics, such as immunotherapy, is another aspect we need to explore in future studies.

## Author Contributions

JL and MS designed and drafted the manuscript. DC participated in revising the manuscript critically. All authors contributed to the article and approved the submitted version.

## Conflict of Interest

The authors declare that the research was conducted in the absence of any commercial or financial relationships that could be construed as a potential conflict of interest.

## Publisher’s Note

All claims expressed in this article are solely those of the authors and do not necessarily represent those of their affiliated organizations, or those of the publisher, the editors and the reviewers. Any product that may be evaluated in this article, or claim that may be made by its manufacturer, is not guaranteed or endorsed by the publisher.

## References

[1] Siegel RL Miller KD Fuchs HE Jemal A. Cancer statistics, 2021. *CA Cancer J Clin.* (2021) 71:7–33. 10.3322/caac.21654 33433946

[2] Fodde R. The Apc gene in colorectal cancer. *Eur J Cancer.* (2002) 38:867–71. 10.1016/s0959-8049(02)00040-0 11978510

[3] Iacopetta B. Tp53 mutation in colorectal cancer. *Hum Mutat.* (2003) 21:271–6. 10.1002/humu.10175 12619112

[4] Porru M Pompili L Caruso C Biroccio A Leonetti C. Targeting kras in metastatic colorectal cancer: current strategies and emerging opportunities. *J Exp Clin Cancer Res.* (2018) 37:57. 10.1186/s13046-018-0719-1 29534749 PMC5850913

[5] Whiteside TL. The tumor microenvironment and its role in promoting tumor growth. *Oncogene.* (2008) 27:5904–12. 10.1038/onc.2008.271 18836471 PMC3689267

[6] Shen M Kang Y. Complex interplay between tumor microenvironment and cancer therapy. *Front Med.* (2018) 12:426–39. 10.1007/s11684-018-0663-7 30097962

[7] Wong SH Yu J. Gut microbiota in colorectal cancer: mechanisms of action and clinical applications. *Nat Rev Gastroenterol Hepatol.* (2019) 16:690–704. 10.1038/s41575-019-0209-8 31554963

[8] Wroblewski LE Peek RM Jr. Wilson KT. *Helicobacter* pylori and gastric cancer: factors that modulate disease risk. *Clin Microbiol Rev.* (2010) 23:713–39. 10.1128/CMR.00011-10 20930071 PMC2952980

[9] Gagniere J Raisch J Veziant J Barnich N Bonnet R Buc E Gut microbiota imbalance and colorectal cancer. *World J Gastroenterol.* (2016) 22:501–18. 10.3748/wjg.v22.i2.501 26811603 PMC4716055

[10] Huycke MM Abrams V Moore DR. *Enterococcus faecalis* produces extracellular superoxide and hydrogen peroxide that damages colonic epithelial cell DNA. *Carcinogenesis.* (2002) 23:529–36. 10.1093/carcin/23.3.529 11895869

[11] Ruiz PA Shkoda A Kim SC Sartor RB Haller D. Il-10 gene-deficient mice lack Tgf-Beta/Smad signaling and fail to inhibit proinflammatory gene expression in intestinal epithelial cells after the colonization with colitogenic *Enterococcus Faecalis*. *J Immunol.* (2005) 174:2990–9. 10.4049/jimmunol.174.5.2990 15728512

[12] Kumar R Herold JL Schady D Davis J Kopetz S Martinez-Moczygemba M *Streptococcus gallolyticus* Subsp. *gallolyticus* promotes colorectal tumor development. *PLoS Pathog.* (2017) 13:e1006440. 10.1371/journal.ppat.1006440 28704539 PMC5509344

[13] Aymeric L Donnadieu F Mulet C du Merle L Nigro G Saffarian A Colorectal cancer specific conditions promote *Streptococcus gallolyticus* gut colonization. *Proc Natl Acad Sci USA.* (2018) 115:E283–91. 10.1073/pnas.1715112115 29279402 PMC5777054

[14] Chen C Mao Y Du J Xu Y Zhu Z Cao H. *Helicobacter* pylori infection associated with an increased risk of colorectal adenomatous polyps in the Chinese population. *BMC Gastroenterol.* (2019) 19:14. 10.1186/s12876-018-0918-4 30665367 PMC6341657

[15] Coelho LGV Coelho MCF. *Helicobacter* pylori and colorectal neoplasms: a concise review. *Arq Gastroenterol.* (2021) 58:114–9. 10.1590/S0004-2803.202100000-19 33909789

[16] Rhee KJ Wu S Wu X Huso DL Karim B Franco AA Induction of persistent colitis by a human commensal, enterotoxigenic *Bacteroides* fragilis, in wild-type C57bl/6 mice. *Infect Immun.* (2009) 77:1708–18. 10.1128/IAI.00814-08 19188353 PMC2663167

[17] Boleij A Hechenbleikner EM Goodwin AC Badani R Stein EM Lazarev MG The *Bacteroides* fragilis toxin gene is prevalent in the colon mucosa of colorectal cancer patients. *Clin Infect Dis.* (2015) 60:208–15. 10.1093/cid/ciu787 25305284 PMC4351371

[18] Dahmus JD Kotler DL Kastenberg DM Kistler CA. The gut microbiome and colorectal cancer: a review of bacterial pathogenesis. *J Gastrointest Oncol.* (2018) 9:769–77. 10.21037/jgo.2018.04.07 30151274 PMC6087872

[19] Raisch J Buc E Bonnet M Sauvanet P Vazeille E de Vallee A Colon cancer-associated B2 *Escherichia Coli* colonize gut mucosa and promote cell proliferation. *World J Gastroenterol.* (2014) 20:6560–72. 10.3748/wjg.v20.i21.6560 24914378 PMC4047342

[20] Raisch J Rolhion N Dubois A Darfeuille-Michaud A Bringer MA. Intracellular colon cancer-associated *Escherichia Coli* promote protumoral activities of human macrophages by inducing sustained Cox-2 expression. *Lab Invest.* (2015) 95:296–307. 10.1038/labinvest.2014.161 25545478

[21] Rubinstein MR Baik JE Lagana SM Han RP Raab WJ Sahoo D *Fusobacterium nucleatum* promotes colorectal cancer by inducing Wnt/Beta-Catenin modulator annexin A1. *EMBO Rep.* (2019) 20:e47638. 10.15252/embr.201847638 30833345 PMC6446206

[22] Okumura S Konishi Y Narukawa M Sugiura Y Yoshimoto S Arai Y Gut bacteria identified in colorectal cancer patients promote Tumourigenesis *via* butyrate secretion. *Nat Commun.* (2021) 12:5674. 10.1038/s41467-021-25965-x 34584098 PMC8479117

[23] Zhou Y Ye C Lou Y Liu J Ye S Chen L Epigenetic mechanisms underlying organic solute transporter beta repression in colorectal cancer. *Mol Pharmacol.* (2020) 97:259–66. 10.1124/mol.119.118216 32005758

[24] Flynn C Montrose DC Swank DL Nakanishi M Ilsley JN Rosenberg DW. Deoxycholic acid promotes the growth of colonic aberrant crypt foci. *Mol Carcinog.* (2007) 46:60–70. 10.1002/mc.20253 17091474

[25] Kim DH Jin YH. Intestinal bacterial beta-glucuronidase activity of patients with colon cancer. *Arch Pharm Res.* (2001) 24:564–7. 10.1007/BF02975166 11794536

[26] Kawee-Ai A Kim SM. Application of microalgal fucoxanthin for the reduction of colon cancer risk: inhibitory activity of fucoxanthin against beta-glucuronidase and Dld-1 cancer cells. *Nat Prod Commun.* (2014) 9:921–4. 25230493

[27] Ambalam P Raman M Purama RK Doble M. Probiotics, prebiotics and colorectal cancer prevention. *Best Pract Res Clin Gastroenterol.* (2016) 30:119–31. 10.1016/j.bpg.2016.02.009 27048903

[28] Yue Y Ye K Lu J Wang X Zhang S Liu L Probiotic strain *Lactobacillus plantarum* Yyc-3 prevents colon cancer in mice by regulating the tumour microenvironment. *Biomed Pharmacother.* (2020) 127:110159. 10.1016/j.biopha.2020.110159 32353824

[29] Chen ZF Ai LY Wang JL Ren LL Yu YN Xu J Probiotics *Clostridium butyricum* and *Bacillus* subtilis ameliorate intestinal tumorigenesis. *Future Microbiol.* (2015) 10:1433–45. 10.2217/fmb.15.66 26346930

[30] Oberreuther-Moschner DL Jahreis G Rechkemmer G Pool-Zobel BL. Dietary intervention with the probiotics *Lactobacillus acidophilus* 145 and *Bifidobacterium longum* 913 modulates the potential of human faecal water to induce damage in Ht29clone19a Cells. *Br J Nutr.* (2004) 91:925–32. 10.1079/BJN20041108 15182396

[31] Blackwood BP Yuan CY Wood DR Nicolas JD Grothaus JS Hunter CJ. Probiotic *Lactobacillus* species strengthen intestinal barrier function and tight junction integrity in experimental necrotizing enterocolitis. *J Probiotics Health.* (2017) 5:159. 10.4172/2329-8901.1000159 28638850 PMC5475283

[32] Lee HA Kim H Lee KW Park KY. Dead Nano-Sized *Lactobacillus plantarum* inhibits Azoxymethane/Dextran Sulfate sodium-induced colon cancer in Balb/C mice. *J Med Food.* (2015) 18:1400–5. 10.1089/jmf.2015.3577 26595186

[33] Pithva SP Ambalam PS Ramoliya JM Dave JM Vyas BR. Antigenotoxic and Antimutagenic activities of probiotic *Lactobacillus rhamnosus* Vc against N-Methyl-N’-Nitro-N-Nitrosoguanidine. *Nutr Cancer.* (2015) 67:1142–50. 10.1080/01635581.2015.1073751 26312410

[34] Verma A Shukla G. Probiotics *Lactobacillus rhamnosus* Gg, *Lactobacillus acidophilus* suppresses Dmh-induced Procarcinogenic Fecal Enzymes and Preneoplastic aberrant crypt foci in early colon carcinogenesis in sprague dawley rats. *Nutr Cancer.* (2013) 65:84–91. 10.1080/01635581.2013.741746 23368917

[35] Wang S Sun J Chen K Ma P Lei Q Xing S Perspectives of tumor-infiltrating lymphocyte treatment in solid tumors. *BMC Med.* (2021) 19:140. 10.1186/s12916-021-02006-4 34112147 PMC8194199

[36] de la Cruz-Merino L Henao Carrasco F Vicente Baz D Nogales Fernandez E Reina Zoilo JJ Codes Manuel de Villena M Immune microenvironment in colorectal cancer: a new hallmark to change old paradigms. *Clin Dev Immunol.* (2011) 2011:174149. 10.1155/2011/174149 22162710 PMC3226426

[37] Wilkinson K Ng W Roberts TL Becker TM Lim SH Chua W Tumour immune microenvironment biomarkers predicting cytotoxic chemotherapy efficacy in colorectal cancer. *J Clin Pathol.* (2021) 74:625–34. 10.1136/jclinpath-2020-207309 33753562 PMC8461409

[38] Huang Z Liu Y Qi G Brand D Zheng SG. Role of vitamin a in the immune system. *J Clin Med.* (2018) 7:258. 10.3390/jcm7090258 30200565 PMC6162863

[39] Qi J Crinier A Escaliere B Ye Y Wang Z Zhang T Single-cell transcriptomic landscape reveals tumor specific innate lymphoid cells associated with colorectal cancer progression. *Cell Rep Med.* (2021) 2:100353. 10.1016/j.xcrm.2021.100353 34467243 PMC8385246

[40] Goc J Lv M Bessman NJ Flamar AL Sahota S Suzuki H Dysregulation of Ilc3s unleashes progression and immunotherapy resistance in colon cancer. *Cell.* (2021) 184:5015–30.e16. 10.1016/j.cell.2021.07.029 34407392 PMC8454863

[41] Huang EY Chang JC Chen HH Hsu CY Hsu HC Wu KL. Carcinoembryonic antigen as a marker of radioresistance in colorectal cancer: a potential role of macrophages. *BMC Cancer.* (2018) 18:321. 10.1186/s12885-018-4254-4 29580202 PMC5870371

[42] Cheng Y Zhu Y Xu W Xu J Yang M Chen P Pkcalpha in colon cancer cells promotes m1 macrophage polarization *via* Mkk3/6-P38 mapk pathway. *Mol Carcinog.* (2018) 57:1017–29. 10.1002/mc.22822 29637628

[43] Goswami KK Ghosh T Ghosh S Sarkar M Bose A Baral R. Tumor promoting role of anti-tumor macrophages in tumor microenvironment. *Cell Immunol.* (2017) 316:1–10. 10.1016/j.cellimm.2017.04.005 28433198

[44] Zhang W Chen L Ma K Zhao Y Liu X Wang Y Polarization of macrophages in the tumor microenvironment is influenced by Egfr signaling within colon cancer cells. *Oncotarget.* (2016) 7:75366–78. 10.18632/oncotarget.12207 27683110 PMC5342747

[45] Edin S Wikberg ML Rutegard J Oldenborg PA Palmqvist R. Phenotypic skewing of macrophages *in vitro* by secreted factors from colorectal cancer cells. *PLoS One.* (2013) 8:e74982. 10.1371/journal.pone.0074982 24058644 PMC3776729

[46] Wu Y Yuan L Lu Q Xu H He X. Distinctive profiles of tumor-infiltrating immune cells and association with intensity of infiltration in colorectal cancer. *Oncol Lett.* (2018) 15:3876–82. 10.3892/ol.2018.7771 29456737 PMC5795874

[47] Gardner A Ruffell B. Dendritic cells and cancer immunity. *Trends Immunol.* (2016) 37:855–65. 10.1016/j.it.2016.09.006 27793569 PMC5135568

[48] Galati D Corazzelli G De Filippi R Pinto A. Dendritic cells in hematological malignancies. *Crit Rev Oncol Hematol.* (2016) 108:86–96.. 10.1016/j.critrevonc.2016.10.006 27931844

[49] Truxova I Kasikova L Hensler M Skapa P Laco J Pecen L Mature dendritic cells correlate with favorable immune infiltrate and improved prognosis in ovarian carcinoma patients. *J Immunother Cancer.* (2018) 6:139. 10.1186/s40425-018-0446-3 30526667 PMC6288908

[50] Li J Yang J Hua L Wang R Li H Zhang C Ese-3 contributes to colon cancer progression by downregulating Ehd2 and transactivating Inpp4b. *Am J Cancer Res.* (2021) 11:92–107. 33520362 PMC7840712

[51] Sprater F Azeem W Appel S. Activation of peroxisome proliferator-activated receptor gamma leads to upregulation of Ese-3 expression in human monocyte-derived dendritic cells. *Scand J Immunol.* (2014) 79:20–6. 10.1111/sji.12126 24219556

[52] Rizzo M Bayo J Piccioni F Malvicini M Fiore E Peixoto E Low molecular weight hyaluronan-pulsed human dendritic cells showed increased migration capacity and induced resistance to tumor chemoattraction. *PLoS One.* (2014) 9:e107944. 10.1371/journal.pone.0107944 25238610 PMC4169605

[53] Triner D Devenport SN Ramakrishnan SK Ma X Frieler RA Greenson JK Neutrophils restrict tumor-associated microbiota to reduce growth and invasion of colon tumors in mice. *Gastroenterology.* (2019) 156:1467–82. 10.1053/j.gastro.2018.12.003 30550822 PMC6441634

[54] Yamamoto M Kikuchi H Ohta M Kawabata T Hiramatsu Y Kondo K Tsu68 prevents liver metastasis of colon cancer xenografts by modulating the premetastatic niche. *Cancer Res.* (2008) 68:9754–62. 10.1158/0008-5472.CAN-08-1748 19047154

[55] Hirai H Fujishita T Kurimoto K Miyachi H Kitano S Inamoto S Ccr1-mediated accumulation of myeloid cells in the liver microenvironment promoting mouse colon cancer metastasis. *Clin Exp Metastasis.* (2014) 31:977–89. 10.1007/s10585-014-9684-z 25326065 PMC4256518

[56] Bhome R Bullock MD Al Saihati HA Goh RW Primrose JN Sayan AE A top-down view of the tumor microenvironment: structure, cells and signaling. *Front Cell Dev Biol.* (2015) 3:33. 10.3389/fcell.2015.00033 26075202 PMC4448519

[57] Herrera M Llorens C Rodriguez M Herrera A Ramos R Gil B Differential distribution and enrichment of non-coding rnas in exosomes from normal and cancer-associated fibroblasts in colorectal cancer. *Mol Cancer.* (2018) 17:114. 10.1186/s12943-018-0863-4 30075793 PMC6091058

[58] Yang X Li Y Zou L Zhu Z. Role of exosomes in crosstalk between cancer-associated fibroblasts and cancer cells. *Front Oncol.* (2019) 9:356. 10.3389/fonc.2019.00356 31131261 PMC6510008

[59] Huang D Sun W Zhou Y Li P Chen F Chen H Mutations of key driver genes in colorectal cancer progression and metastasis. *Cancer Metastasis Rev.* (2018) 37:173–87. 10.1007/s10555-017-9726-5 29322354

[60] Zhang Y Wang S Lai Q Fang Y Wu C Liu Y Cancer-associated fibroblasts-derived exosomal Mir-17-5p promotes colorectal cancer aggressive phenotype by initiating a Runx3/Myc/Tgf-Beta1 positive feedback loop. *Cancer Lett.* (2020) 491:22–35. 10.1016/j.canlet.2020.07.023 32730779

[61] Gaggioli C Hooper S Hidalgo-Carcedo C Grosse R Marshall JF Harrington K Fibroblast-led collective invasion of carcinoma cells with differing roles for rhogtpases in leading and following cells. *Nat Cell Biol.* (2007) 9:1392–400. 10.1038/ncb1658 18037882

[62] Otomo R Otsubo C Matsushima-Hibiya Y Miyazaki M Tashiro F Ichikawa H Tspan12 is a critical factor for cancer-fibroblast cell contact-mediated cancer invasion. *Proc Natl Acad Sci USA.* (2014) 111:18691–6. 10.1073/pnas.1412062112 25512506 PMC4284611

[63] Semba S Kodama Y Ohnuma K Mizuuchi E Masuda R Yashiro M Direct cancer-stromal interaction increases fibroblast proliferation and enhances invasive properties of scirrhous-type gastric carcinoma cells. *Br J Cancer.* (2009) 101:1365–73. 10.1038/sj.bjc.6605309 19773759 PMC2768433

[64] Zadka L Chabowski M Grybowski D Piotrowska A Dziegiel P. Interplay of stromal tumor-infiltrating lymphocytes, normal colonic mucosa, cancer-associated fibroblasts, clinicopathological data and the iommunoregulatory molecules of patients diagnosed with colorectal cancer. *Cancer Immunol Immunother.* (2021) 70:2681–700. 10.1007/s00262-021-02863-1 33625532 PMC8360892

[65] Schellerer VS Langheinrich M Hohenberger W Croner RS Merkel S Rau TT Tumor-associated fibroblasts isolated from colorectal cancer tissues exhibit increased icam-1 expression and affinity for monocytes. *Oncol Rep.* (2014) 31:255–61. 10.3892/or.2013.2860 24253852

[66] Zhang R Qi F Zhao F Li G Shao S Zhang X Cancer-associated fibroblasts enhance tumor-associated macrophages enrichment and suppress Nk cells function in colorectal cancer. *Cell Death Dis.* (2019) 10:273. 10.1038/s41419-019-1435-2 30894509 PMC6426970

[67] Nagasaki T Hara M Nakanishi H Takahashi H Sato M Takeyama H. Interleukin-6 released by colon cancer-associated fibroblasts is critical for tumour angiogenesis: anti-interleukin-6 receptor antibody suppressed angiogenesis and inhibited tumour-stroma interaction. *Br J Cancer.* (2014) 110:469–78. 10.1038/bjc.2013.748 24346288 PMC3899773

[68] Erdogan B Ao M White LM Means AL Brewer BM Yang L Cancer-associated fibroblasts promote directional cancer cell migration by aligning fibronectin. *J Cell Biol.* (2017) 216:3799–816. 10.1083/jcb.201704053 29021221 PMC5674895

[69] Walker C Mojares E Del Rio Hernandez A. Role of extracellular matrix in development and cancer progression. *Int J Mol Sci.* (2018) 19:3028. 10.3390/ijms19103028 30287763 PMC6213383

[70] De Wever O Nguyen QD Van Hoorde L Bracke M Bruyneel E Gespach C Tenascin-C and Sf/Hgf produced by myofibroblasts *in vitro* provide convergent pro-invasive signals to human colon cancer cells through Rhoa and Rac. *FASEB J.* (2004) 18:1016–8. 10.1096/fj.03-1110fje 15059978

[71] Bauer J Emon MAB Staudacher JJ Thomas AL Zessner-Spitzenberg J Mancinelli G Increased stiffness of the tumor microenvironment in colon cancer stimulates cancer associated fibroblast-mediated prometastatic activin a signaling. *Sci Rep.* (2020) 10:50. 10.1038/s41598-019-55687-6 31919369 PMC6952350

[72] Nishida N Yano H Nishida T Kamura T Kojiro M. Angiogenesis in cancer. *Vasc Health Risk Manag.* (2006) 2:213–9. 10.2147/vhrm.2006.2.3.213 17326328 PMC1993983

[73] Zetter BR. Angiogenesis and tumor metastasis. *Annu Rev Med.* (1998) 49:407–24. 10.1146/annurev.med.49.1.407 9509272

[74] St Croix B Rago C Velculescu V Traverso G Romans KE Montgomery E Genes expressed in human tumor endothelium. *Science.* (2000) 289:1197–202. 10.1126/science.289.5482.1197 10947988

[75] Lu J Ye X Fan F Xia L Bhattacharya R Bellister S Endothelial cells promote the colorectal cancer stem cell phenotype through a soluble form of jagged-1. *Cancer Cell.* (2013) 23:171–85. 10.1016/j.ccr.2012.12.021 23375636 PMC3574187

[76] Nubel T Dippold W Kleinert H Kaina B Fritz G. Lovastatin inhibits rho-regulated expression of e-selectin by Tnfalpha and attenuates tumor cell adhesion. *FASEB J.* (2004) 18:140–2. 10.1096/fj.03-0261fje 14630701

[77] Motz GT Santoro SP Wang LP Garrabrant T Lastra RR Hagemann IS Tumor endothelium fasl establishes a selective immune barrier promoting tolerance in tumors. *Nat Med.* (2014) 20:607–15. 10.1038/nm.3541 24793239 PMC4060245

[78] Wang J Uddin MN Akter R Wu Y. Contribution of endothelial cell-derived transcriptomes to the colon cancer based on bioinformatics analysis. *Math Biosci Eng.* (2021) 18:7280–300. 10.3934/mbe.2021360 34814249

[79] Hu L Hayashi Y Kidoya H Takakura N. Endothelial cell-derived apelin inhibits tumor growth by altering immune cell localization. *Sci Rep.* (2021) 11:14047. 10.1038/s41598-021-93619-5 34234274 PMC8263715

[80] Naschberger E Liebl A Schellerer VS Schutz M Britzen-Laurent N Kolbel P Matricellular protein sparcl1 regulates tumor microenvironment-dependent endothelial cell heterogeneity in colorectal carcinoma. *J Clin Invest.* (2016) 126:4187–204. 10.1172/JCI78260 27721236 PMC5096916

[81] Khawar IA Kim JH Kuh HJ. Improving drug delivery to solid tumors: priming the tumor microenvironment. *J Control Release.* (2015) 201:78–89. 10.1016/j.jconrel.2014.12.018 25526702

[82] Shoulders MD Raines RT. Collagen structure and stability. *Annu Rev Biochem.* (2009) 78:929–58. 10.1146/annurev.biochem.77.032207.120833 19344236 PMC2846778

[83] Xu S Xu H Wang W Li S Li H Li T The role of collagen in cancer: from bench to bedside. *J Transl Med.* (2019) 17(1):309. 10.1186/s12967-019-2058-1 31521169 PMC6744664

[84] Wu X Cai J Zuo Z Li J. Collagen facilitates the colorectal cancer stemness and metastasis through an integrin/Pi3k/Akt/Snail signaling pathway. *Biomed Pharmacother.* (2019) 114:108708. 10.1016/j.biopha.2019.108708 30913493

[85] Wang J Jiang YH Yang PY Liu F. Increased collagen type V Alpha2 (Col5a2) in colorectal cancer is associated with poor prognosis and tumor progression. *Onco Targets Ther.* (2021) 14:2991–3002. 10.2147/OTT.S288422 33981148 PMC8107053

[86] Yi W Xiao E Ding R Luo P Yang Y. High expression of fibronectin is associated with poor prognosis, cell proliferation and malignancy *via* the Nf-Kappab/P53-apoptosis signaling pathway in colorectal cancer. *Oncol Rep.* (2016) 36:3145–53. 10.3892/or.2016.5177 27748871 PMC5112592

[87] Ye Y Zhang R Feng H. Fibronectin promotes tumor cells growth and drugs resistance through a Cdc42-yap-dependent signaling pathway in colorectal cancer. *Cell Biol Int.* (2020) 44:1840–9. 10.1002/cbin.11390 32437085

[88] Najafi M Farhood B Mortezaee K. Extracellular matrix (Ecm) stiffness and degradation as cancer drivers. *J Cell Biochem.* (2019) 120:2782–90. 10.1002/jcb.27681 30321449

[89] Tan F Huang Y Pei Q Liu H Pei H Zhu H. Matrix stiffness mediates stemness characteristics *via* activating the yes-associated protein in colorectal cancer cells. *J Cell Biochem.* (2019) 120:2213–25. 10.1002/jcb.27532 30218452

[90] Liu C Pei H Tan F. Matrix stiffness and colorectal cancer. *Onco Targets Ther.* (2020) 13:2747–55. 10.2147/OTT.S231010 32280247 PMC7131993

[91] Ikeda K Iyama K Ishikawa N Egami H Nakao M Sado Y Loss of expression of Type Iv collagen Alpha5 and Alpha6 chains in colorectal cancer associated with the hypermethylation of their promoter region. *Am J Pathol.* (2006) 168:856–65. 10.2353/ajpath.2006.050384 16507901 PMC1606532

[92] Sala M Ros M Saltel FA. Complex and evolutive character: two face aspects of Ecm in tumor progression. *Front Oncol.* (2020) 10:1620. 10.3389/fonc.2020.01620 32984031 PMC7485352

[93] Becker C Fantini MC Schramm C Lehr HA Wirtz S Nikolaev A Tgf-beta suppresses tumor progression in colon cancer by inhibition of il-6 trans-signaling. *Immunity.* (2004) 21:491–501. 10.1016/j.immuni.2004.07.020 15485627

[94] Bellam N Pasche B. Tgf-beta signaling alterations and colon cancer. *Cancer Treat Res.* (2010) 155:85–103. 10.1007/978-1-4419-6033-7_5 20517689

[95] Schatoff EM Leach BI Dow LE. Wnt signaling and colorectal cancer. *Curr Colorectal Cancer Rep.* (2017) 13(2):101–10. 10.1007/s11888-017-0354-9 28413363 PMC5391049

[96] Ogata-Kawata H Izumiya M Kurioka D Honma Y Yamada Y Furuta K Circulating exosomal micrornas as biomarkers of colon cancer. *PLoS One.* (2014) 9:e92921. 10.1371/journal.pone.0092921 24705249 PMC3976275

[97] Danac JMC Uy AGG Garcia RL. Exosomal micrornas in colorectal cancer: overcoming barriers of the metastatic cascade (Review). *Int J Mol Med.* (2021) 47:112. 10.3892/ijmm.2021.4945 33907829 PMC8075282

[98] Sun LH Tian D Yang ZC Li JL. Exosomal Mir-21 promotes proliferation, invasion and therapy resistance of colon adenocarcinoma cells through its target Pdcd4. *Sci Rep.* (2020) 10:8271. 10.1038/s41598-020-65207-6 32427870 PMC7237414

[99] Clancy C Khan S Glynn CL Holian E Dockery P Lalor P Screening of exosomal micrornas from colorectal cancer cells. *Cancer Biomark.* (2016) 17:427–35. 10.3233/CBM-160659 27802194 PMC13020523

[100] Ochiai T Masuda T Yagi M Kasai R Furuyama T Tsukamoto K Successful combination therapy of radical liver resection with 5-Fluorouracil/Leucovorin, Oxaliplatin, Plus Bevacizumab for ascending colon cancer with pulmonary and 43 liver metastases: report of a case. *Int Surg.* (2012) 97:6–13. 10.9738/CC88.1 23101994 PMC3723196

[101] Galizia G Lieto E Orditura M Castellano P Imperatore V Pinto M First-line chemotherapy vs bowel tumor resection plus chemotherapy for patients with unresectable synchronous colorectal hepatic metastases. *Arch Surg.* (2008) 143:352–8; discussion 358. 10.1001/archsurg.143.4.352. 18427022

[102] Zeelenberg IS Ruuls-Van Stalle L Roos E. The chemokine receptor Cxcr4 is required for outgrowth of colon carcinoma micrometastases. *Cancer Res.* (2003) 63:3833–9. 12839981

[103] Wang R Bhattacharya R Ye X Fan F Boulbes DR Ellis LM. Endothelial cells promote colorectal cancer cell survival by activating the Her3-Akt pathway in a paracrine fashion. *Mol Cancer Res.* (2019) 17:20–9. 10.1158/1541-7786.MCR-18-0341 30131447 PMC6318043

[104] Harmon C Robinson MW Hand F Almuaili D Mentor K Houlihan DD Lactate-mediated acidification of tumor microenvironment induces apoptosis of liver-resident nk cells in colorectal liver metastasis. *Cancer Immunol Res.* (2019) 7:335–46. 10.1158/2326-6066.CIR-18-0481 30563827

[105] Shao Y Chen T Zheng X Yang S Xu K Chen X Colorectal cancer-derived small extracellular vesicles establish an inflammatory premetastatic niche in liver metastasis. *Carcinogenesis.* (2018) 39:1368–79. 10.1093/carcin/bgy115 30184100

[106] Jiang K Chen H Fang Y Chen L Zhong C Bu T Exosomal angptl1 attenuates colorectal cancer liver metastasis by regulating Kupffer cell secretion pattern and impeding Mmp9 induced vascular leakiness. *J Exp Clin Cancer Res.* (2021) 40:21. 10.1186/s13046-020-01816-3 33413536 PMC7792106

[107] Mitry E Guiu B Cosconea S Jooste V Faivre J Bouvier AM. Epidemiology, management and prognosis of colorectal cancer with lung metastases: a 30-year population-based study. *Gut.* (2010) 59:1383–8. 10.1136/gut.2010.211557 20732912

[108] Luo JL Maeda S Hsu LC Yagita H Karin M. Inhibition of Nf-Kappab in cancer cells converts inflammation- induced tumor growth mediated by Tnfalpha to trail-mediated tumor regression. *Cancer Cell.* (2004) 6:297–305. 10.1016/j.ccr.2004.08.012 15380520

[109] Zeng Z Li Y Pan Y Lan X Song F Sun J Cancer-derived exosomal Mir-25-3p promotes pre-metastatic niche formation by inducing vascular permeability and angiogenesis. *Nat Commun.* (2018) 9:5395. 10.1038/s41467-018-07810-w 30568162 PMC6300604

[110] Ji Q Zhou L Sui H Yang L Wu X Song Q Primary tumors release Itgbl1-rich extracellular vesicles to promote distal metastatic tumor growth through fibroblast-niche formation. *Nat Commun.* (2020) 11:1211. 10.1038/s41467-020-14869-x 32139701 PMC7058049

[111] Nadler A McCart JA Govindarajan A. Peritoneal carcinomatosis from colon cancer: a systematic review of the data for cytoreduction and intraperitoneal chemotherapy. *Clin Colon Rectal Surg.* (2015) 28:234–46. 10.1055/s-0035-1564431 26648794 PMC4655111

[112] van Gestel YR de Hingh IH van Herk-Sukel MP van Erning FN Beerepoot LV Wijsman JH Patterns of metachronous metastases after curative treatment of colorectal cancer. *Cancer Epidemiol.* (2014) 38:448–54. 10.1016/j.canep.2014.04.004 24841870

[113] Mo S Cai G. Multidisciplinary treatment for colorectal peritoneal metastases: review of the literature. *Gastroenterol Res Pract.* (2016) 2016:1516259. 10.1155/2016/1516259 28105045 PMC5220469

[114] Lemoine L Sugarbaker P Van der Speeten K. Pathophysiology of colorectal peritoneal carcinomatosis: role of the peritoneum. *World J Gastroenterol.* (2016) 22:7692–707. 10.3748/wjg.v22.i34.7692 27678351 PMC5016368

[115] Wronski M Arbit E. Resection of brain metastases from colorectal carcinoma in 73 patients. *Cancer.* (1999) 85:1677–85. 10.1002/(sici)1097-0142(19990415)85:83.0.co;2-c 10223560

[116] Murata J Ricciardi-Castagnoli P Dessous L’Eglise Mange P Martin F Juillerat-Jeanneret L. Microglial cells induce cytotoxic effects toward colon carcinoma cells: measurement of tumor cytotoxicity with a gamma-glutamyl transpeptidase assay. *Int J Cancer.* (1997) 70:169–74. 10.1002/(sici)1097-0215(19970117)70:23.0.co;2-v 9009156

[117] Murata J Corradin SB Janzer RC Juillerat-Jeanneret L. Tumor cells suppress cytokine-induced nitric-oxide (no) production in cerebral endothelial cells. *Int J Cancer.* (1994) 59:699–705. 10.1002/ijc.2910590519 7525497

[118] Challa VR Goud YG Rangappa P Deshmane V Kumar KV Madhusudhana BA. Ovarian metastases from colorectal cancer: our experience. *Indian J Surg Oncol.* (2015) 6:95–8. 10.1007/s13193-014-0369-5 26405412 PMC4577483

[119] Yamanishi Y Koshiyama M Ohnaka M Ueda M Ukita S Hishikawa K Pathways of metastases from primary organs to the ovaries. *Obstet Gynecol Int.* (2011) 2011:612817. 10.1155/2011/612817 21915181 PMC3170892

[120] Kubecek O Laco J Spacek J Petera J Kopecky J Kubeckova A The pathogenesis, diagnosis, and management of metastatic tumors to the ovary: a comprehensive review. *Clin Exp Metastasis.* (2017) 34:295–307. 10.1007/s10585-017-9856-8 28730323 PMC5561159

[121] Geller LT Barzily-Rokni M Danino T Jonas OH Shental N Nejman D Potential role of intratumor bacteria in mediating tumor resistance to the chemotherapeutic drug gemcitabine. *Science.* (2017) 357:1156–60. 10.1126/science.aah5043 28912244 PMC5727343

[122] Nooij S Ducarmon QR Laros JFJ Zwittink RD Norman JM Smits WK Fecal microbiota transplantation influences procarcinogenic *Escherichia Coli* in recipient recurrent clostridioides difficile patients. *Gastroenterology.* (2021) 161:1218–28.e5. 10.1053/j.gastro.2021.06.009 34126062

[123] Xie YH Chen YX Fang JY. Comprehensive review of targeted therapy for colorectal cancer. *Signal Transduct Target Ther.* (2020) 5:22. 10.1038/s41392-020-0116-z 32296018 PMC7082344

[124] Waldman AD Fritz JM Lenardo MJA. Guide to cancer immunotherapy: from t cell basic science to clinical practice. *Nat Rev Immunol.* (2020) 20:651–68. 10.1038/s41577-020-0306-5 32433532 PMC7238960

[125] Dai Y Zhao W Yue L Dai X Rong D Wu F Perspectives on immunotherapy of metastatic colorectal cancer. *Front Oncol.* (2021) 11:659964. 10.3389/fonc.2021.659964 34178645 PMC8219967

[126] Loeffler M Kruger JA Niethammer AG Reisfeld RA. Targeting tumor-associated fibroblasts improves cancer chemotherapy by increasing intratumoral drug uptake. *J Clin Invest.* (2006) 116:1955–62. 10.1172/JCI26532 16794736 PMC1481657

[127] Li M Li M Yin T Shi H Wen Y Zhang B Targeting of cancerassociated fibroblasts enhances the efficacy of cancer chemotherapy by regulating the tumor microenvironment. *Mol Med Rep.* (2016) 13:2476–84. 10.3892/mmr.2016.4868 26846566 PMC4768992

[128] Qi L Song F Han Y Zhang Y Ding Y. Atractyloside targets cancer-associated fibroblasts and inhibits the metastasis of colon cancer. *Ann Transl Med.* (2020) 8:1443. 10.21037/atm-20-1531 33313188 PMC7723590

[129] Sakurai Y Akita H Harashima H. Targeting tumor endothelial cells with nanoparticles. *Int J Mol Sci.* (2019) 20:5819. 10.3390/ijms20235819 31756900 PMC6928777

[130] Shen M Jiang YZ Wei Y Ell B Sheng X Esposito M Tinagl1 suppresses triple-negative breast cancer progression and metastasis by simultaneously inhibiting integrin/Fak and Egfr signaling. *Cancer Cell.* (2019) 35:64–80.e7. 10.1016/j.ccell.2018.11.016 30612941

[131] Stoeltzing O Liu W Reinmuth N Fan F Parry GC Parikh AA Inhibition of integrin alpha5beta1 function with a small peptide (Atn-161) plus continuous 5-fu infusion reduces colorectal liver metastases and improves survival in mice. *Int J Cancer.* (2003) 104:496–503. 10.1002/ijc.10958 12584749

[132] Liu Z Ren Y Pan L Xu HM. *In vivo* anti-tumor activity of polypeptide hm-3 modified by different polyethylene glycols (Peg). *Int J Mol Sci.* (2011) 12:2650–63. 10.3390/ijms12042650 21731464 PMC3127140

[133] Dai S Wei D Wu Z Zhou X Wei X Huang H Phase I clinical trial of autologous ascites-derived exosomes combined with gm-csf for colorectal cancer. *Mol Ther.* (2008) 16:782–90. 10.1038/mt.2008.1 18362931 PMC7106337

[134] Hanahan D. Hallmarks of cancer: new dimensions. *Cancer Discov.* (2022) 12:31–46. 10.1158/2159-8290.CD-21-1059 35022204

[135] Gupta GP Massague J. Cancer metastasis: building a framework. *Cell.* (2006) 127:679–95. 10.1016/j.cell.2006.11.001 17110329

[136] Celia-Terrassa T Kang Y. Distinctive properties of metastasis-initiating cells. *Genes Dev.* (2016) 30:892–908. 10.1101/gad.277681.116 27083997 PMC4840296

[137] Shen M Kang Y. Stresses in the metastatic cascade: molecular mechanisms and therapeutic opportunities. *Genes Dev.* (2020) 34:1577–98. 10.1101/gad.343251.120 33262145 PMC7706714

